# Spin filtering effect generated by the inter-subband spin-orbit coupling in the bilayer nanowire with the quantum point contact

**DOI:** 10.1038/srep45346

**Published:** 2017-03-30

**Authors:** Paweł Wójcik, Janusz Adamowski

**Affiliations:** 1 AGH University of Science and Technology, Faculty of Physics and Applied Computer Science, al. Mickiewicza 30, Kraków, Poland

## Abstract

The spin filtering effect in the bilayer nanowire with quantum point contact is investigated theoretically. We demonstrate the new mechanism of the spin filtering based on the lateral inter-subband spin-orbit coupling, which for the bilayer nanowires has been reported to be strong. The proposed spin filtering effect is explained as the joint effect of the Landau-Zener intersubband transitions caused by the hybridization of states with opposite spin (due to the lateral Rashba SO interaction) and the confinement of carriers in the quantum point contact region.

A fabrication of a controllable source of a spin polarized current that operates without a magnetic field is one of the most important challenges of semiconductor spintronics^1^. Among spin filters proposed over the years, including these based on carbon nanotubes^2^, quantum dots^3^, Y-shaped nanostructures^4^,^5^ or resonant tunneling diodes^6^,^7^,^8^, recently, the special attention is paid to the quantum point contacts (QPC) with the spin-orbit (SO) interaction^9^,^10^,^11^,^12^,^13^,^14^,^15^,^16^,^17^. In such nanostructures, the spin filtering effect results from the interplay between the SO coupling^18^,^19^ and the quantum confinement. Recent papers^9^,^10^,^11^,^12^,^13^,^14^,^15^ have reported the experimental evidence of the spin filtering in QPCs, which manifests itself as the plateau of conductance at 0.5*G*_0_ (*G*_0_ = 2*e*^2^/h) measured in the absence of the external magnetic field. The 0.5*G*_0_ plateau has been explained as resulting from the combination of the three effects^13^,^14^,^17^: an asymmetric lateral confinement, a lateral Rashba SO interaction, and an electron - electron interaction. More precisely, the asymmetry in a lateral confinement, induced by the different voltages applied to the side electrodes of QPC, is a source of a lateral electric field. Due to the SO interaction, this electric field, in the electron’s rest frame, is seen as an effective magnetic field, which initializes an imbalance between the spin-up and spin-down electrons. As shown by Ngo *et al*.^17^ this so-called lateral Rashba SO interaction leads to the low spin polarization of the current not exceeding 6%, and therefore its presence did not explain the 0.5*G*_0_ plateau observed in the experiments. The full explanation has been given by the further studies, which have shown that the predicted weak spin filter effect^17^ can be strengthened by the electron-electron interaction leading to the nearly 100% spin polarization in the regime of the single-mode transport^13^. QPCs with the SO interaction have been successfully used as the spin injector and detector in the recent experimental realization of the spin transistor^20^,^21^, in which about 10^5^ times greater conductance oscillations have been observed as compared to the conventional spin-field effect transistor based on ferromagnets^22^,^23^.

The experimental realizations of spin filters based on QPCs, reported so far, are based on a two-dimensional electron gas (2DEG) confined in the narrow quantum well at the AlGaAs/GaAs or InAs/InAlAs interface^10^,^11^, in which the electrons occupy the lowest-energy state (“single occupancy”). However, recently, wider or coupled quantum wells with two populated subbands (“double occupancy”) have attracted a growing interest of both experimentalists^24^,^25^,^26^,^27^,^28^,^29^ and theoreticians^30^,^31^,^32^,^33^,^34^,^35^. In this case, we deal with the vertically coupled nanowires, in which the coupling strength is determined by the wave function overlap between the ground and first excited states. The additional orbital degree of freedom in the bilayer nanowires leads to interesting physical effects such as inter-subband induced band anticrossing and spin mixing^26^. The SO interaction in quantum well with double occupancy has been studied by Bernardes *et al*.^31^. The inter-subband induced SO interaction has been found which results from the coupling between states with opposite parity. It can give raise to intriguing physical phenomena, e.g. unusual Zitterbewegung^31^ or intrinsic spin Hall effect in symmetric quantum well^27^. The influence of the inter-subband SO interaction in bilayer nanowire on the spin transistor action has been studied in our recent paper^36^. We have shown that the resonant behavior of spin-orbit coupling constants obtained for zero gate voltage leads to the spin transistor operation, in which the on/off transition should be realized in the narrow voltage range.

In the present paper, we demonstrate the novel mechanism of spin filtering based on the lateral inter-subband spin-orbit coupling in the bilayer nanowire with QPC. We find that for the non-zero inter-subband coupling induced by the lateral Rashba SO interaction, the spin polarization of the current flowing through the QPC is almost full. We analyze the conditions, under which this polarization takes place. The observed spin filtering effect is explained as the joint effect of the Landau-Zener inter-subband transitions caused by the hybridization of states with opposite spins and the quantum confinement in the QPC region. Our results provide a new mechanism to implement spin-polarized electron sources in the realistic bilayer nanowires which can be built from the double quantum well or wide quantum well structure.

## Theoretical Model

### Model of the nanostructure

**Figure 1:**
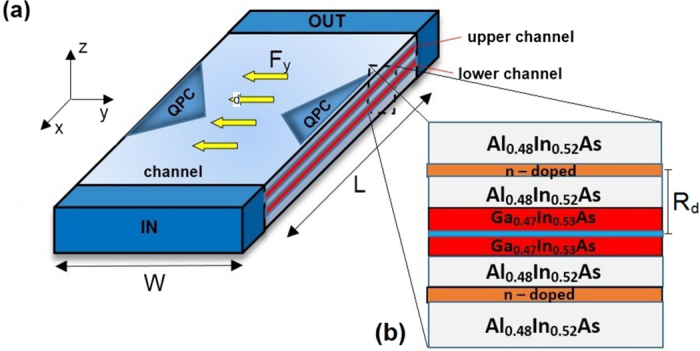
(**a**) Schematic of the bilayer nanowire with the QPC. In the presence of the lateral electric field *F*_*y*_, the unpolarized current injected from the contact IN, after passing through the QPC, is almost fully spin polarized. (b) Cross section of the exemplary realization of the bilayer nanowire based on the Al_0.48_In_0.52_As/Ga_0.47_In_0.53_As double quantum wells.

We consider the bilayer nanowire consisting of two coupled conducting channels of width *W* and length *L* (Fig. 1). Both ends of the nanowire are connected to the reflectionless, ideal leads denoted as IN and OUT. In the middle of the nanowire, the QPC is located as schematically presented in Fig. 1(a). In recent experiments^24^,^25^,^26^,^27^,^28^,^29^, vertically stacked and coupled nanowires with QPC’s are prepared from 2DEG bilayer systems built from double quantum wells or a wide quantum well in which the weak Coulomb repulsion gives rise to the “soft” barrier in the middle of the quantum well. Figure 1(b) presents the cross-section of the exemplary double quantum well heterostructure, which consists of two Al_0.48_In_0.52_As/Ga_0.47_In_0.53_ As quantum wells with a central Al_0.3_In_0.7_ As barrier with width *w*_*b*_, which controls the coupling between the conduction electron states in the quantum wells. For the sufficiently high central barrier, the quantum wells are separated and the electron wave functions are localized in one of the quantum wells. For the symmetric structure each of the states is fourfold degenerate whereby the twofold degeneracy results from the spin states and twofold degeneracy is related to the geometric symmetry. If the height of the barrier decreases, the states in the quantum wells become coupled to each other with the coupling strength determined by the wave function overlap. The formation of the symmetric and antisymmetric (bounding and anti-bounding) states leads to the splitting of the previously degenerate quantum states with the splitting energy in the range 1–10 meV^24^,^25^. For appropriate electron density only the two spin degenerate subbands (the ground and first excited) are occupied (double occupancy) leaving the rest of the higher-energy subbands unoccupied since their energy is considerably higher than the energy of the ground and first excited states.

The spin filtering effect proposed in this paper requires the lateral Rashba SO interaction i.e. SOI generated by the lateral electric field **F** = (0, *F*_*y*_, 0). In fact, recently, Gvozdic and Ekenberg^37^ pointed out that in the modulation-doped wide or coupled quantum wells, used for the bilayer nanowires fabrication, the large intrinsic *F*_*y*_ exists. Alternatively, in the experiment, the lateral electric field can also be generated by the side gates attached to the channel. Regardless of the origin, the lateral electric field *F*_*y*_ induces the Rashba SO interaction with the effective magnetic field directed along the grown *z*-axis. The possibility of using the SO interaction induced by the lateral electric field has been recently reported in many experiments^9^,^10^,^20^, in which the lateral SO coupling constant has been reported to vary in the range 0.04 eVÅ−0.5 eVÅ.

### Numerical methods

Let us define the four element basis 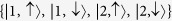 which consists of the spin-degenerate ground and first excited eigenstate related to the confinement in the *z* direction. In the presence of the SO interaction, we can derive the 4 × 4 Hamiltonian in the basis of these states^30^,^36^. After introducing a set **τ** of Pauli-like matrices in the orbital space, the Hamiltonian of the system takes on the form (full derivation of Hamiltonian (1) can be found in Supplementary)


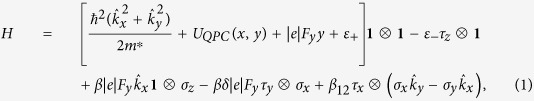


where **1** is the 2 × 2 unit matrix, m^*^ is the electron effective mass, 
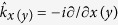
 is the wave vector operator, *β* is the lateral Rashba spin-orbit coupling constant, *β*_12_ is the inter-subband lateral Rashba spin-orbit coupling constant, 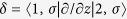 is the inter-subband coupling constant, 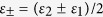 with *ε*_1(2)_ being the energy of eigenstate 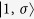 (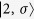), 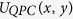 is the potential energy of electron from the QPC which is given by


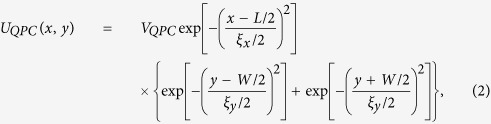


where 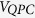 is the maximal potential energy in the QPC while *ξ*_*x*_ and *ξ*_*y*_ determine the extension of the QPC in the *x* and *y* directions, respectively. In Hamiltonian (1) we neglect the intra-subband SO coupling assuming that the system is symmetric in the *z* direction with respect to the reflection z → −*z*^36^. Since the Dresselhaus SO coupling constant 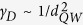 (*d*_*QW*_ is the width of the quantum well in the *z* direction), for the wide quantum well used in the experimental realization of bilayer nanowires, the strength of the Dresselhaus SO interaction is a few orders of magnitude smaller than the Rashba SO coupling. This allows us to neglect the Dresselhaus term in the Hamiltonian (1) - detailed discussion of the Dresselhaus SO coupling in the considered nanostructure and its influence on the presented spin filtering can be found in Supplementary material.

The calculations of the conductance have been performed by the scattering matrix method using the Kwant package^38^. For this purpose we have transformed the Hamiltonian (1) into the discretized form on the grid 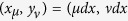) with *μ, ν* = 1, 2, …, where *dx* is the lattice constant. We introduce the discrete representation of the electron state in the 4 × 4 space as follows: 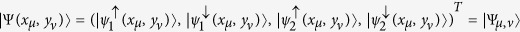. The Hamiltonian (1) takes on the discretized two-dimensional form


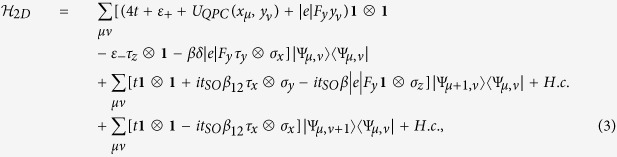


where 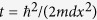 and 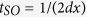.

In the calculations we assume the hard-wall boundary conditions in the *y* direction i.e. 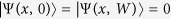. In the *x*-direction, the boundary conditions are set based on the fact that the electron state in the input is a linear combination of the state in which the electron is injected into the channel and all possible states in which the electron can be reflected from the QPC. Therefore


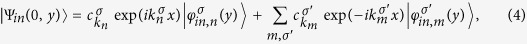


where *n, m* = 1, 2, 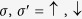 and 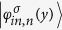 are the eigenstates of the hamiltonian (3) calculated by the use of the boundary conditions in the input (*μ* = 0)


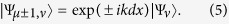


Accordingly, the boundary conditions for the output are given by


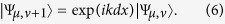


Let us assume that the electron with spin *σ* in the subband *n (n* = 1, 2) is injected from the input contact into the nanowire. The electron can be transmitted through the QPC in one of the four possible processes (in parenthesis the symbol of the transmission probability is given): intra-subband transmission with spin conservation 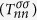, intra-subband transmission with spin-flip 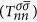, inter-subband transmission with spin conservation (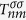), and inter-subband transmission with spin flip (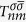), where 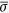 denotes the spin opposite to is *σ*, while 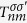 is the probability of the electron transmission between the subbands 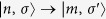, (*n, m* = 1, 2, 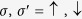).

Having determined the transmission coefficients 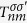 we calculate the conductance in the ballistic regime using the Landauer formula


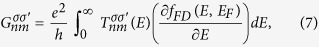


where 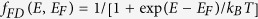 is the Fermi-Dirac distribution function, *T* is the temperature and *E*_*F*_ is the Fermi energy. The spin-dependent conductances through the nanostructure are given by


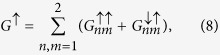



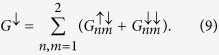


The total conductance 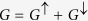 and the spin polarization of current


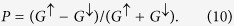


The conductance calculations have been performed for *dx* = 2 nm, *L* = 3000 nm, *W* = 92 nm, *ξ*_*x*_ = 300 nm, *ξ*_*y*_ = 48 nm and 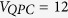 meV. We use the material parameters corresponding to In_0.5_Ga_0.5_As, i.e., *m*_*e*_ = 0.0465*m*_0_ and the Rashba spin-orbit interaction constant 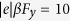 meVnm^10^. The energy difference between the two occupied subbands is taken to be Δ*ε* = *ε*_2_ − *ε*_1_ = 1 meV.

## Results and Discussion

**Figure 2:**
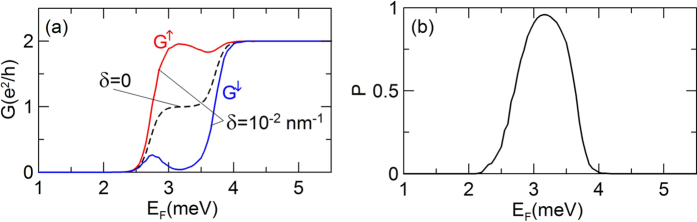
(**a**) Conductance *G* as a function of Fermi energy *E*_*F*_ for two values of inter-subband coupling constants *δ*. Red and blue curves correspond to 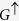 and 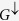, respectively. Black dashed curve shows the results for *δ* = 0, for which 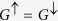. (**b**) Spin polarization *P* of the current as a function of Fermi energy *E*_*F*_ for *δ* =10^−2^ nm^−1^.

**Figure 3:**
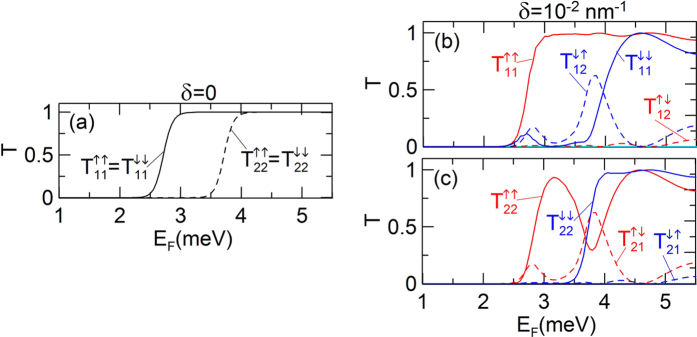
Transmission probabilities 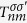 versus Fermi energy *E*_*F*_ for (**a**) *δ* = 0 and (**b,c**) *δ* =10^−2^ nm^−2^ for the electron injected into (**b**) the first subband 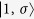 and (**c**) the second subband 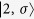, 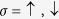.

In this section, we present the results of the conductance calculations and explain the physical mechanism responsible for the nearly full spin polarization obtained in the bilayer nanowires with QPC. First, we assume that the inter-subband induced SO coupling constant *β*_12_ = 0. Figure 2(a) displays the conductance 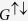 as a function of the Fermi energy *E*_*F*_ for two values of the inter-subband coupling constant *δ*. Red and blue curves correspond to 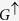 and 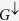, respectively. For *δ* *=* 0, depicted by the black dashed curve, 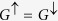 hence the spin polarization of current *P* = 0 in entire range of the Fermi energy. The two conductance steps are due to the subsequent subbands passing through the Fermi level in the QPC region, each contributing to the increase of the conductance by *e*^2^/*h*. For the nonzero inter-subband coupling, i.e. for *δ* = 10^−2^ nm^−1^, the conductances 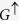 and 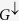 differ from each other in some range of the Fermi energy, which leads to the almost full spin polarization of current presented in Fig. 1(b). We note that the spin polarization occurs only in the Fermi energy range, which corresponds to the conductance step for *δ* *=* 0. The transmission probabilities 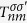 for *δ* *=* 0 and *δ* *=* 10^−2^ nm^−1^ are presented in Fig. 3. Comparing results in Fig. 3(a,b and c) we see that the imbalance between the spin-up and spin-down conductance, in the Fermi energy range, in which the spin polarization is observed, results from the suppression of the intra-subband transmission of spin-down electrons in the first subband 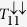 and the enhancement of the intra-subband transmission of spin-up electrons 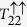. As expected the inter-subband transmissions reveal the symmetry 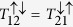 and 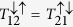.

**Figure 4:**
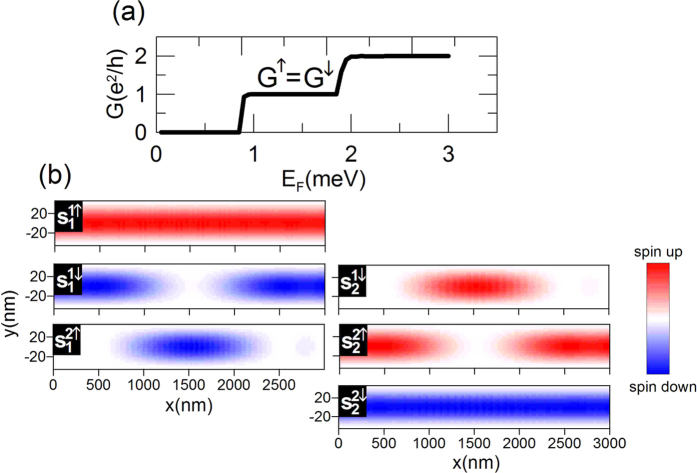
(**a**) Conductance *G* as a function of Fermi energy *E*_*F*_ for 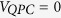. (**b**) The *z* component of the partial spin density distributions 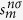, where *m* is the index of the subband, for which the spin density distribution is presented, while *nσ* denotes the index of the subband, including spin, from which the electrons are injected into the nanowire. Results for 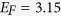 meV and 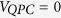.

In order to explain the physical mechanism behind the spin filtering effect let us first consider the nanowire without QPC (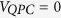). As shown in Fig. 4(a), which presents the spin-dependent conductance 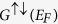, the constriction in the form of QPC located in the nanowire is necessary to obtain the spin filtering. Without QPC, 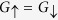, which leads to the spin polarization *P* = 0. The full understanding of the spin filtering mechanism, which emerges when we add QPC, requires the understanding of the spin dynamics in the nanowire without the constriction. Figure 4(b) presents the *z* component of the partial spin density distributions 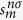, where *m* is the index of the subband, for which the spin density distribution is presented, while *nσ* denotes the index of the subband, including spin, from which the electrons are injected into the nanowire. Explicitly,


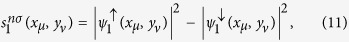



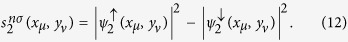


**Figure 5:**
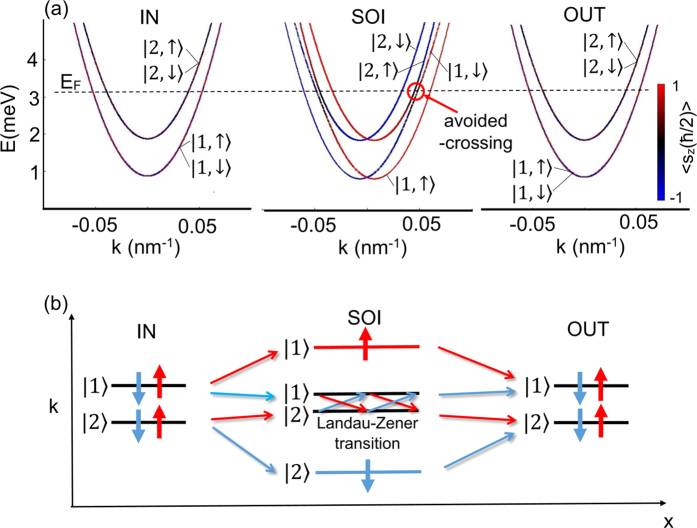
(**a**) Dispersion relations *E* vs *k* in the leads IN and OUT as well as in the nanowire with the SO interaction. The dashed horizontal line denotes the Fermi energy *E*_*F*_ = 3.15 meV, for which the spin density distribution maps are presented in Fig. 4(b). (**b**) Schematic illustration of the possible transmission processes through the nanowire without QPC.

The dispersion relations for the leads IN and OUT as well as in the central part of the nanowire with the SO interaction are presented in Fig. 5(a). The Fermi energy, for which the maps from Fig. 4(b) have been calculated, is marked by the dashed horizontal line. We set *E*_*F*_ = 3.15 meV which corresponds to the maximal spin polarization of current presented in Fig. 2(b).

As shown in Fig. 4(b) the electrons injected in the states 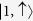 and 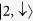 flow through the nanowire in the subband into which they were injected, conserving their spin. Different behavior is observed for the electrons injected in the states 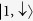 and 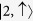. The electrons injected into the subband 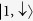 (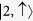) are transmitted to the state 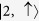 (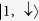) in the middle of the nanowire and are back in their original state before leaving the nanowire through the lead OUT. To explain this behavior let us consider the electron propagating in the state 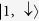 from the input channel IN. In the nanowire where the lateral Rashba SO interaction is present, the spin is no longer a good quantum number, since the orbital and spin degrees of freedom are mixed. For the appropriate Fermi energy the states 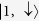 and 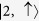 hybridize giving raise to the avoided crossing in the spin-split subbands presented in Fig. 5(a). The probability of the transition through the avoided crossing depends on the degree of the adiabaticity of the electron transport and the avoided crossing width as predicted by the Landau-Zener theory^39^,^40^. For the fully diabatic transport the probability of electron transfer from the state 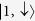 to 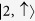 is equal to 1. Therefore, the transfer probability through the avoiding crossing depends on the rate of the energy level changes when the electron from the contact, in which the SO interaction is absent, flows through the nanowires with the SO interaction. In our case, this effect is implemented by the spatially dependent SO coupling constants which vary in accordance with the function


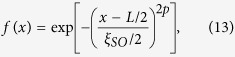


where 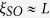 and *p* is the so-called “softness” parameter. We assume *p* = 10, which guarantees the diabatic transport between the leads and the nanowire.

The schematic illustration of all possible electron transmission processes through the nanowire in the *k*-space is presented in Fig. 5(b). Among them the Landau-Zener transition between the states 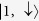 and 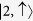 (with spin flip) is a key to understanding the spin filtering effect, which emerges when we introduce the QPC.

**Figure 6:**
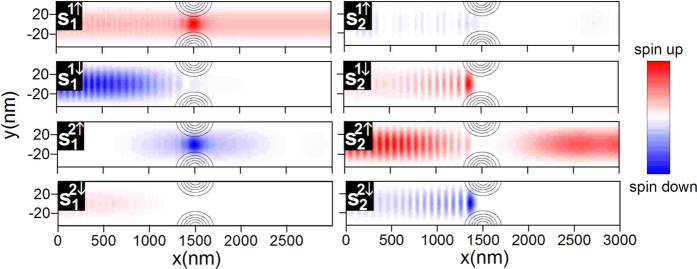
The *z* component of the partial spin density distributions 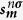, where *m* is the index of the subband in which the spin density distribution is presented, while *nσ* denote the index of the subband, including spin, from which the electrons are injected into the nanowire. The gray contours present the QPC region. Results for *E*_*F*_ = 3.15 meV and *V*_*QPC*_ = 12 meV.

Now, let us explain in detail the spin filtering mechanism, which emerges if we add the QPC. For this purpose, in Fig. 6 we present the *z* component of the partial spin density distribution for 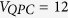 meV. Even a cursory analysis of this figure indicates, that for the chosen Fermi energy, the current through the QPC is carried mainly by the spin-up electrons transmitted through both the subbands, in agreement with the spin-dependent transmission probabilities presented in Fig. 3. For completeness, we have also calculated the local density of states (LDOS).

**Figure 7:**
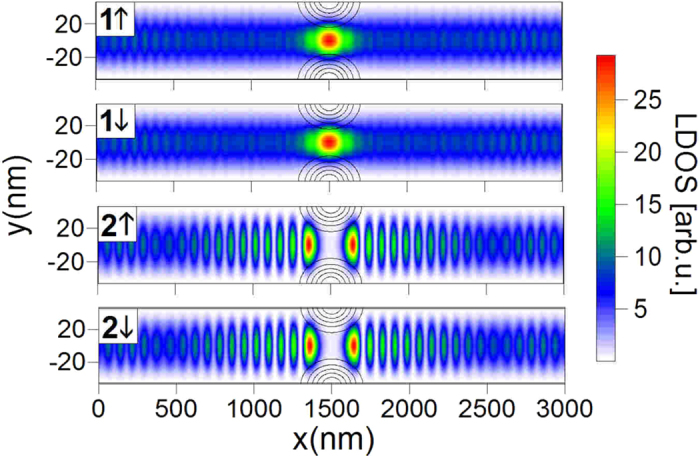
Local density of states (LDOS) calculated for the subbands participating in the electron transport. The gray contours correspond to the QPC region.

**Figure 8:**
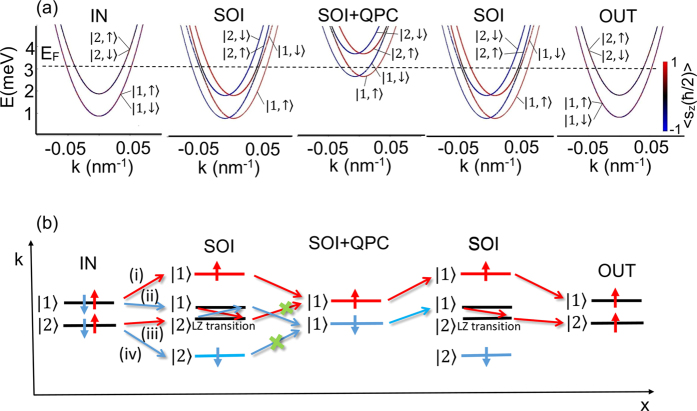
(**a**) Dispersion relation *E(k*) in the leads IN and OUT as well as in the nanowire with the SO interaction and in the QPC region. The dashed horizontal line denotes the Fermi energy for which the spin density distribution maps are presented in Fig. 6. (**b**) Schematic illustration of the possible transmission processes through the nanowire.

Figure 7 shows that there are no available states 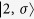 (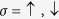) in the QPC region, which means that the electrons reaching QPC in the state 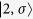, independently of spin, are reflected from it. Now, we consider separately the transport processes for the electrons injected into the nanowire from the subsequent subbands (i) 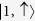, (ii) 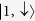, (iii) 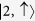 and (iv) 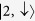. Our analysis will be conducted on the basis of the partial spin density distributions (Figs 2 and 6), LDOS (Fig. 7) and the dispersion relations in different parts of the nanowire presented in Fig. 8(a).

First, we focus on the processes (i) and (iv). As shown in Fig. 4(b), which presents the spin dynamics in the nanowire without QPC, the electrons injected in the state 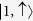 flow through the nanowire conserving their state. Due to the large density of states 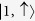 in the QPC region (see Fig. 7), the electrons injected in this subband are transmitted through the QPC giving raise to the spin-up polarized current in the output (see Fig. 6). (iv) Although the electrons in the state 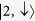 injected into the nanowire without QPC also flow through the nanowire conserving the state [Fig. 4(b)], if we introduce the QPC, the electrons are backscattered from the constriction due to the lack of the states 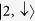 in the QPC region as presented in Fig. 7.

The spin dynamics occurring for the electrons injected in the states 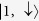 and 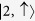 is much more complicated. (ii) Due to the hybridization of the subbands 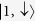 and 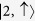 induced by the SO interaction (see avoiding crossing in Fig. 8(a)), before reaching the QPC the electrons injected in the state 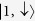 are transmitted to the state 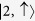. This process is similar to the previously explained spin dynamics, which occur in the nanowire without QPC [Fig. 4(b)]. Therefore, when reaching the QPC the electrons initially injected in 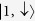 are in the state 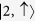 for which LDOS is zero in the QPC region (Fig. 7). These electrons are backscattered from the QPC as presented in Fig. 6. The enhancement of the spin polarization of current is mainly related to the electrons injected in the state 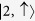 (iii). Although there are no available states for the subband 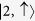 in the QPC region (Fig. 7), the electrons in the subband 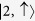 injected from the contact IN into the nanowire are transmitted to the state 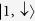 before reaching the QPC (see Fig. 6). The Landau-Zener transition is possible due to the lateral SO interaction, which causes the hybridization of the states 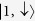 and 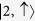. Since LDOS in the QPC region for the state 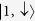 is large (Fig. 7), the electrons pass through the QPC and, as show in Fig. 6, just after passing through the QPC they are transmitted to the state 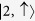 leaving the nanowire in this subband. This process, together with the direct transition through the QPC in the state 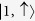 result in the nearly full spin-up polarization of the current for the chosen Fermi energy. The transport precesses (i)–(iv) are schematically illustrated in Fig. 8(b).

**Figure 9:**
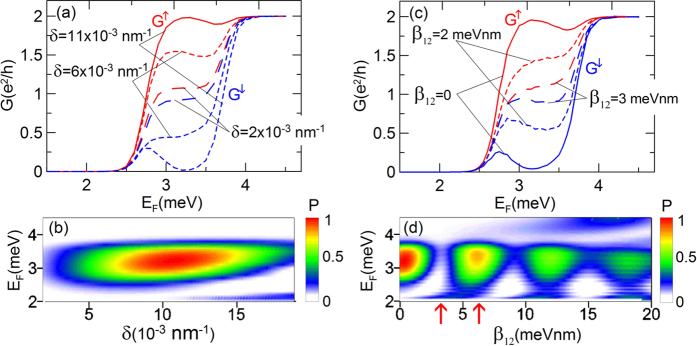
(**a**) Conductance 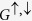 as a function of Fermi energy *E*_*F*_ for different values of inter-subband coupling constant *δ*. Red and blue lines correspond to 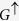 and 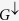, respectively. (**b**) Spin polarization of current *P* as a function of Fermi energy *E*_*F*_ and inter-subband coupling constant *δ*. (**c**) Conductance 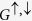 as a function of Fermi energy *E*_*F*_ for different values of inter-subband induced SO coupling constant *β*_12_ and *δ* = 10^−2^ nm^−1^. (**d**) Spin polarization of current *P* as a function of Fermi energy *E*_*F*_ and inter-subband induced SO coupling constant *β*_12_. The values of *β*_12_ marked by the red arrows are taken to the further analysis. Results for 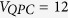 meV.

Based on the above analysis one can determine three important factors necessary to achieve the spin polarization of current in the bilayer nanowires: (a) the lateral Rashba SO interaction, (b) the inter-subband coupling and (c) the constriction, which in our case has the form of the QPC. Among these factors the inter-subband coupling resulting from the lateral Rashba SO interaction plays the crucial role. In order to show how the value of the parameter *δ* affects the spin polarization of current, in Fig. 9(a) we present 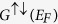 for different value of *δ*. The map of the spin polarization *P* as a function of the Fermi energy *E*_*F*_ and the inter-subband coupling constant *δ* is presented in Fig. 9(b). These results show that there is a value of *δ*, for which the spin polarization is the largest and reaches *P* = 1.

**Figure 10:**
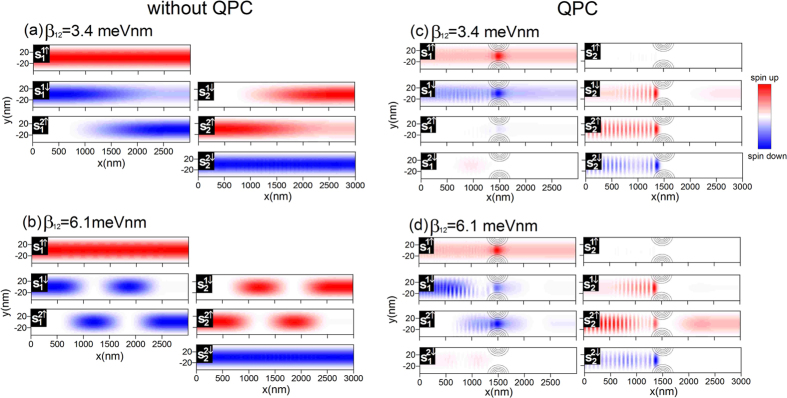
The *z* component of the partial spin density distributions 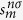 calculated for the nanowire without QPC (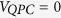), for (**a**) *β*_12_ = 3.4 meVnm and (**b**) *β*_12_ = 6.1 meVnm and for the nanowire with QPC (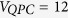 meV), for (**c**) *β*_12_ = 3.4 meVnm and (**d**) *β*_12_ = 6.1 meVnm. The gray contours in figures (**c**) and (**d**) correspond to the QPC region. Results for *E*_*F*_ = 3.15 meV.

All the results presented so far have been obtained for the inter-subband SO coupling constant *β*_12_ = 0. However, the recent theoretical studies^30^, in which the self-consistent calculations of the intra- and inter-subband SO coupling constants were performed for the double quantum wells, reported that the inter-subband SO coupling constant exhibits the resonant behavior reaching the values comparable to the ordinary (intra-subband) Rashba SO constant. For this reason we introduce the inter-subband SO interaction to our model. Figure 9(c,d) presents the conductance 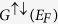 for different values of the inter-subband SO coupling constants *β*_12_ assuming *δ* = 10^−2^ nm^−1^. The dependence of the spin polarization *P* on *E*_*F*_ and *β*_12_ presented in Fig. 9(d) reveals the damped oscillations of *P* as a function of *β*_12_ with the period approximately equal to 6 meVnm. The inter-subband SO interaction causes that the electrons flowing through the nanostructure are periodically transfered between the hybridized states 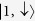 and 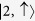. Each of these transitions takes place at a specific distance, that depends on the coupling constant *β*_12_. Depending of the number of transitions, which occur when the electrons pass the distance from the lead IN to the QPC region we obtain the maximal (for odd number of transitions) or minimal (for even number of transitions) spin polarization. In order to illustrate this property, in Fig. 10 we present the *z* component of the partial spin density distributions 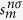 for two chosen inter-subband induced SO coupling constants: (a) *β*_12_ = 3.4 meVnm corresponding to the minimum and (b) *β*_12_ = 6.1 meVnm corresponding to the maximum of *P*. The chosen values of *β*_12_ are marked by the red arrows in Fig. 9(d). Since our goal is to analyze the inter-subband spin dynamics under the influence of the inter-subband SO interaction and its impact on the spin filtering effect, results in Fig. 10 are presented for the two cases, for the nanowire without QPC (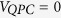) (a, b) and with QPC (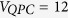 meV) (c, d).

Comparing the spin dynamics for the nanowire without QPC presented in Figs 4 and 10(a,b) one can conclude that the only difference, which appears when we include the inter-subband induced SO interaction, is the distance that the electron injected in the state 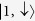 (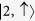) needs to make a transition to the state 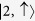 (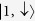). Moreover, independently of the inter-subband SO coupling 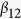, the electrons injected in the states 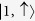 and 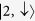 remain in their states flowing through the nanowire. As explained, the spin filtering mechanism presented in the paper requires that the electrons injected from the contact IN in state 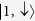 (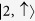) make transitions to the other state and reach the QPC region in state 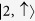 (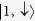). This necessary condition causes that the electrons injected in the state 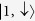 are backscattered from the QPC while the electrons in the state 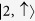 pass through the QPC giving raise to the high spin polarization of the current. As shown in Fig. 10(a), for *β*_12_ = 3.4 meVnm corresponding to the spin polarization minima, the electrons in the state 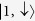 (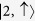) are transmitted to the state 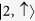 (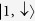) after traveling the distance *L*. Since the QPC is located at *x* = L/2, the electrons injected from the lead IN reach the QPC in the state that is mostly built from the initial state. This means that the spin filtering mechanism described in the paper will not occur. Instead, as presented in Fig. 10(c) the electrons in the states 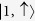 and 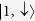 simply pass through the QPC while the electrons in the states 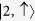 and 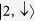 are backscattered due to zero LDOS in the QPC region (see Fig. 7). The electron transport in the nanowire through the subbands with opposite spin leads to the spin polarization nearly equal to zero, i.e. the unpolarized current. The further increase of the inter-subband induced SO interaction constant causes that the distance over which the electron are transmitted between the states 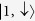 and 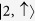 becomes shorter. For *β*_12_ = 6.1 meVnm (Fig. 10(b) and (d)) the necessary condition, for which the spin filtering effect occurs, namely the inter-subband transition before reaching QPC, is again satisfied giving raise to the high spin polarization of the current. We can summarize that depending on the number of the inter-subband transitions, which occur before the electron reaches the QPC acting as the channel selector, the spin polarization oscillates between the large and small values as presented in Fig. 9(d).

**Figure 11:**
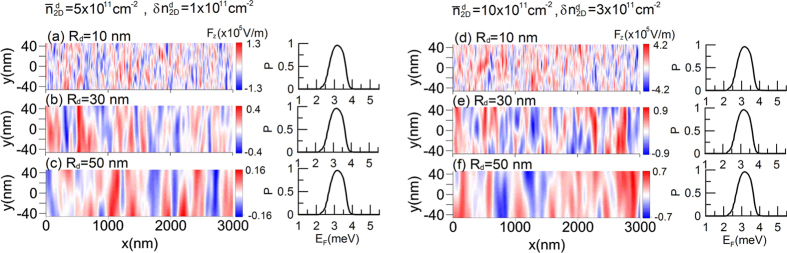
Spatial distributions of the electric field 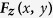 calculated for the different 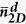 and 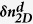 as well as different distances *R*_*d*_ of the dopant layers from the main quantum well. Right panels present the spin polarization of the current *P(E*_*F*_) calculated with the inclusion of the random component of the spin-orbit coupling generated by the electric field distribution presented on the left panels.

Our results in general provide a new mechanism to implement spin-polarized electron sources in the bilayer nanowires. Therefore, it is important to discuss this proposal from the point of view of the possible perturbations, which can appear in the experimental realization of the proposed spin filter. Our model assumes three necessary conditions, which have to be satisfied in order to achieve the high spin polarization of the current: (a) the lateral Rashba SO interaction, (b) the inter-subband coupling and (c) the constriction, which in the present paper has the form of the QPC. The lateral Rashba SO interaction is the most important requirement because it generates the coupling between the subbands, which in the consider nanostructures, is crucial for the spin polarization. However, in the wells made of materials with the zincblende crystallographic structure (GaAs, InAs, etc.), the SO coupling also originates from the bulk Dresselhaus term. Although in the GaAs-based structures both the Rashba and Dresselhaus terms are often of the same order of magnitudes, in the InGaAs-based wells growing in the [001] direction, the Rashba term dominates. Since the linear Dresselhaus parameter 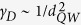, this domination is strengthened for the wide quantum wells needed for the experimental realization of the bilayer nanowires, e.g for the gated Al_0.48_In_0.52_As/Ga_0.47_In_0.53_ As double quantum well structure, the Dresselhaus SO parameter is a four orders of magnitude smaller than the Rashba coupling constant - see the Supplementary material. We have checked by performing direct calculations that the inclusion of such a small term into our model does not affect the spin filtering presented in the paper. Although the neglect of the Dresselhaus term in our model is fully justified, the assumption of constant, spatially independent Rashba parameter requires a detailed discussion. In the considerations presented so far, we assume that the Rashba SO parameter is constant and spatially independent. However, in the realistic nanostructure any local imperfection leads to the local change in the SO coupling. Since, the carriers in the quantum well come from donors, the electric field of ionized donors generates the random, spatially dependent electrostatic potential in the quantum well. This causes that the SO interaction has a random component, which for some structures can be large. Such strong fluctuations of the Rashba coupling constant have been recently measured by the scanning tunnelling microscopy in the structure with 2DEG fabricated by adsorbing Cs on the p-type InSb(110)^41^. In that case^41^, the strong spatial fluctuations of the Rashba parameter between 0.4 eVÅ and 1.6 eVÅ result from the particular, random locations of the dopant ions, which are very close to the inversion layer where the electron density is located. In the Al_0.48_In_0.52_As/Ga_0.47_In_0.53_ As double quantum well structure suggested to the experimental realization of the proposed spin filter, the donor-doped layers are located symmetrically on both sides of the well in the distance *R*_*d*_ much larger than the their width *w*_*d*_ (usually *R*_*d*_/*w*_*d*_ ≈ 5–10).The random distribution of the electric field and the Rashba spin-orbit parameter in the main quantum well originating from the inhomogeneity of the dopant concentration in the donor-doped layers has been discussed in detail in ref. 42. Following this model^42^ we have studied the role of the random spin-orbit coupling component generated by the donors on the spin filter effect presented in the paper. For this purpose, we have calculated the *z*-component of the electric field of the dopant ions with the local concentration 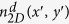



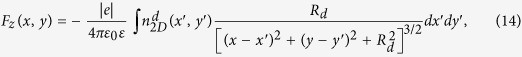


where *e* is the electron charge and *ε* is the dielectric constant. We assume that the random distribution of dopants 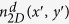 obeys the Gaussian statistics with the mean value 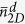 and the standard deviation 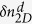. In Fig. 11 we present the spatial distributions of the electric field 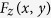 calculated for the different mean values of donor concentration 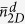 and deviations 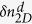 as well as different distances *R*_*d*_ of the dopant layers from the main quantum well. We assume *ε* = 14 corresponding to InAs. The spatially dependent electric field *F*_*z*_(*x, y*) leads to the random distribution of the intra-subband SO coupling *β*_11_ and *β*_22_ [see hamiltonian (5) in the Supplementary material] which in the presented model were assumed to be zero due to the 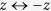 symmetry. In order to show how this effect affects the spin-filtering in the considered nanostructures, we include the random component of the spin-orbit coupling into our model and calculate the spin polarization of the current *P* as a function of the Fermi energy *E*_*F*_. The right panels in Fig. 11 present the spin polarization of the current *P(E*_*F*_) calculated with the inclusion of the spatial distribution of the intra-subband spin-orbit parameters, generated by the electric field distribution presented on the left panels. In the calculations we assume 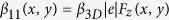 (we take on 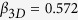 nm^−2^ corresponding to InAs) and 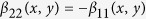. The second assumption is based on our recent results^36^ which show that the intra-subband spin-orbit coupling constants for the ground and first excited state have nearly the same value but opposite sign. The rest of the parameters are assumed to be the same as used in the calculations presented the Fig. 2. Figure 11 shows that for the reasonable values of the dopant concentration and its location with respect to the main quantum well, the random spatial distribution of the spin-orbit coupling does not affect the spin filtering presented in the paper. Its negligible contribution results from the fact that the *z*-component of the electric field of the ionised dopants is several orders of magnitude smaller than the lateral electric field needed to obtain the spin filter effect and used in the experiments for modulation of the Rashba parameter by the QPCs^10^. In the above discussion we did not consider the screening of the *z*-component of the electric field due to the 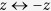 symmetry^42^. As shown in ref. 42, the screening is important for the in-plane components of the electric field, however, the estimated ratio of the fluctuations of the lateral and transversal components of the electric field^42^ in the symmetrically doped quantum well is 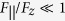. Therefore, the screening strongly reduces the fluctuations of the lateral electric field and (as much smaller than the transversal one) does not affect the spin filter effect presented in the paper.

## Summary

The inter-subband SO interaction attracts the growing interest, because it gives raise to interesting physical effects, e.g., unusual Zitterbewegung^31^. In the bilayer nanowires, the strength of this specific SO interaction, arising from the coupling between states with opposite parity, is comparable to the Rashba intra-subband SO coupling. It makes the bilayer nanowires a good candidate for investigating the inter-subband SO interaction and the effects related with it.

In the present paper, we have proposed the spin filtering mechanism based on the inter-subband SO interaction in the bilayer nanowire with QPC. For this purpose we have studied the electron transport through the nanowire within the two-subband model including the inter-subband SO interaction induced by the lateral electric field. We have found that for the non-zero inter-subband coupling, in the presence of the lateral Rashba SO interaction, the current flowing through the QPC is almost fully spin polarized. In order to explain the spin filtering effect, first we have considered the bilayer nanowire without QPC. By the use of the partial spin density distributions calculated for each of the subband participating in the transport, we have shown that the electrons injected in the state 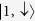 (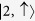) are transmitted to the state 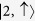 (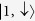) in the middle of the nanowire and again are back in their original state before leaving the nanowire. On the other hand, the electrons in the states 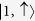 and 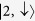 flow through the nanowire conserving their state. The observed Landau-Zener transitions are caused by the hybridization of states 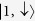 and 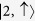 induced by the lateral Rashba SO interaction, which mixes the orbital and spin degrees of freedom. The proposed spin filtering mechanism emerges after adding the QPC, which blocks the electron traveling in the states 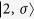 - as shown in Fig. 7, LDOS for these states in the QPC region is zero. Therefore, the introduction of the QPC causes that only the electrons injected into the states 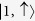 and 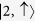 are transmitted through the QPC giving raise to the high spin polarization of the current - the electrons in the state 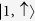 pass through the QPC remaining in their state while the electrons injected in 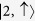 are transmitted to the state 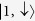 before reaching QPC, pass through the QPC in the state 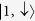 and just behind the QPC are again transmitted to 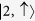 leaving the nanowire in this state. Summing up, the proposed spin filtering effect can be explained as the combined effect of the Landau-Zener inter-subband transitions caused by the hybridization of states with opposite spin and the confinement in the QPC region. We have determined the three important factors necessary to achieve the high spin polarization of the current in the bilayer nanowire: (a) the lateral Rashba SO interaction, (b) the inter-subband coupling and (c) the constriction which in our case has the form of the QPC.

Our results in general provide a new mechanism to implement spin-polarized electron sources in the realistic bilayer nanowires with QPC, which can be realized experimentally in the double quantum well structure or the wide quantum well. This is especially interesting in the context of the current research on the spin filtering in QPC with single occupancy^9^,^10^,^11^,^12^,^13^,^14^,^15^. In those systems, the lateral Rashba SO interaction causes a small spin imbalance, which then is gained by the electron-electron interaction. Our proposal, based on the inter-subband SO interaction in the bilayer nanowires, gives the nearly full spin polarization even without inclusion of the electron-electron interaction. This allows us to expect that the proposed spin filtering effect is more efficient and in the near future can lead to the fabrication of the efficient spin filter.

## Additional Information

**How to cite this article:** Wójcik, P. and Adamowski, J. Spin filtering effect generated by the inter-subband spin-orbit coupling in the bilayer nanowire with the quantum point contact. *Sci. Rep.* **7**, 45346; doi: 10.1038/srep45346 (2017).

**Publisher's note:** Springer Nature remains neutral with regard to jurisdictional claims in published maps and institutional affiliations.

## Supplementary Material

Supplementary Information
