# Supplementary material for: Spin filtering effect generated by the inter-subband spin-orbit coupling in the bilayer nanowire with the quantum point contact
Source: Sci Rep. 2017 Mar 30;7:45346. doi: 10.1038/srep45346 (PMC5371906; doi:10.1038/srep45346)
Supplement: Supplementary Information [file srep45346-s1.pdf]

Supplementary material

Spin filtering effect generated by the inter-subband spin-orbit coupling in the bilayer nanowire with the quantum point contact

Paweł Wójcik[1], Janusz Adamowski[1]
[1] AGH University of Science and Technology, Faculty of Physics and Applied Computer Science, al. Mickiewicza 30, Kraków, Poland

Effective 2D Hamitonian in the two-subband model with the intra- and inter-subband SO interaction

The 3D Hamiltonian of the conduction band electron in the nanowire has the form

$$H_{3D} = -\frac{\hbar^2}{2m^*}\nabla^2 + U(x,y,z) + H_{SO}, \tag{1}$$

where m^* is the conduction band effective mass and the potential energy

$$U(x,y,z) = U_{QPC}(x,y) + |e|F_y y + U_{conf}(z) \tag{2}$$

is the sum of the electron energy in the QPC (U_{QPC}), in the lateral electric field (second term) and the confinement potential energy along the grown direction z, which includes the band potential energy profile and the Hartree energy that describes the electron-electron interaction. H_{SO} in (1) is the Hamiltonian of the Rashba SO interaction, which is given by

$$H_{SO} = \beta \boldsymbol{\sigma} \cdot \left(\nabla U \times \hat{\mathbf{k}}\right), \tag{3}$$

where $\boldsymbol{\sigma} = (\sigma_x, \sigma_y, \sigma_z)$ is the Pauli matrices vector, β is the Rashba spin-orbit coupling constant and $\mathbf{k}$ is the wave vector. Now, we derive the effective 2D Hamiltonian in the two-subband model starting from its 3D form given by Eq. (1). For this purpose we define the four element basis $\{|\varphi_1\rangle|\uparrow\rangle, |\varphi_1\rangle|\downarrow\rangle, |\varphi_2\rangle|\uparrow\rangle, |\varphi_2\rangle|\downarrow\rangle\}$ which consists of the spin-degenerate ground (φ_1) and first excited (φ_2) eigenstates of the Hamiltonian

$$H = -\frac{\hbar^2}{2m^*}\frac{d^2}{dz^2} + U_{conf}(z). \tag{4}$$

The matrix elements, including spin, are given by

$$\begin{aligned}
\langle\varphi_1|H_{3D}|\varphi_1\rangle &= -\frac{\hbar^2}{2m^*}\nabla^2_{2D} + U_{QPC}(x,y) + |e|F_y y + \varepsilon_1 + \beta\left\langle\varphi_1\left|\frac{dU_{conf}(z)}{dz}\right|\varphi_1\right\rangle(\sigma_x\hat{k}_y - \sigma_y\hat{k}_x) \\
&+ \beta\sigma_z\left[\left(\frac{\partial U_{QPC}(x,y)}{\partial y} + |e|F_y\right)\hat{k}_x - \frac{\partial U_{QPC}(x,y)}{\partial x}\hat{k}_y\right], \\
\langle\varphi_1|H_{3D}|\varphi_2\rangle &= \beta\left\langle\varphi_1\left|\frac{dU_{conf}(z)}{dz}\right|\varphi_2\right\rangle(\sigma_x\hat{k}_y - \sigma_y\hat{k}_x) + i\beta\left\langle\varphi_1\left|\frac{d}{dz}\right|\varphi_2\right\rangle\left[\sigma_x\left(\frac{\partial U_{QPC}(x,y)}{\partial y} + |e|F_y\right) - \sigma_y\frac{\partial U_{QPC}(x,y)}{\partial x}\right], \\
\langle\varphi_2|H_{3D}|\varphi_1\rangle &= \beta\left\langle\varphi_1\left|\frac{dU_{conf}(z)}{dz}\right|\varphi_2\right\rangle(\sigma_x\hat{k}_y - \sigma_y\hat{k}_x) - i\beta\left\langle\varphi_1\left|\frac{d}{dz}\right|\varphi_2\right\rangle\left[\sigma_x\left(\frac{\partial U_{QPC}(x,y)}{\partial y} + |e|F_y\right) - \sigma_y\frac{\partial U_{QPC}(x,y)}{\partial x}\right], \\
\langle\varphi_2|H_{3D}|\varphi_2\rangle &= -\frac{\hbar^2}{2m^*}\nabla^2_{2D} + U_{QPC}(x,y) + |e|F_y y + \varepsilon_2 + \beta\left\langle\varphi_2\left|\frac{dU_{conf}(z)}{dz}\right|\varphi_2\right\rangle(\sigma_x\hat{k}_y - \sigma_y\hat{k}_x) \\
&+ \beta\sigma_z\left[\left(\frac{\partial U_{QPC}(x,y)}{\partial y} + |e|F_y\right)\hat{k}_x - \frac{\partial U_{QPC}(x,y)}{\partial x}\hat{k}_y\right].
\end{aligned}$$

If we define the intra-subband SO coupling constants $\beta_{11} = \langle\varphi_1|dU_{conf}(z)/dz|\varphi_1\rangle$ and $\beta_{22} = \langle\varphi_2|dU_{conf}(z)/dz|\varphi_2\rangle$, the inter-subband SO coupling constant $\beta_{12} = \langle\varphi_1|dU_{conf}(z)/dz|\varphi_2\rangle$, the inter-subband coupling constant $\delta = \langle\varphi_1|d/dz|\varphi_2\rangle$, and we

neglect the electric field of the QPC, as negligibly small compared with the lateral electric field ($\partial U_{QPC}/\partial x, \partial U_{QPC}/\partial y \ll F_y$) we obtain

$$H_{2D} = \begin{pmatrix} H_0+\varepsilon_1+\beta|e|F_y\hat{k}_x & \beta_{11}(\hat{k}_y+i\hat{k}_x) & 0 & \beta_{12}(\hat{k}_y+i\hat{k}_x)+i\beta\delta|e|F_y \\ \beta_{11}(\hat{k}_y-i\hat{k}_x) & H_0+\varepsilon_1-\beta|e|F_y\hat{k}_x & \beta_{12}(\hat{k}_y-i\hat{k}_x)+i\beta\delta|e|F_y & 0 \\ 0 & \beta_{12}(\hat{k}_y+i\hat{k}_x)-i\beta\delta|e|F_y & H_0+\varepsilon_2+\beta|e|F_y\hat{k}_x & \beta_{22}(\hat{k}_y+i\hat{k}_x) \\ \beta_{12}(\hat{k}_y-i\hat{k}_x)-i\beta\delta|e|F_y & 0 & \beta_{22}(\hat{k}_y-i\hat{k}_x) & H_0+\varepsilon_2-\beta|e|F_y\hat{k}_x \end{pmatrix}, \quad (5)$$

where

$$H_0 = -\frac{\hbar^2}{2m^*}\nabla^2_{2D} + U_{QPC}(x,y) + |e|F_y y. \quad (6)$$

Assuming $\beta_{11} = \beta_{22} = 0$ we obtain the effective Hamiltonian used in the present paper

$$H = \left[\frac{\hbar^2(\hat{k}_x^2+\hat{k}_y^2)}{2m^*} + U_{QPC}(x,y) + |e|F_y y + \varepsilon_+\right]\mathbf{1}\otimes\mathbf{1} - \varepsilon_-\tau_z\otimes\mathbf{1} + \beta|e|F_y\hat{k}_x\mathbf{1}\otimes\sigma_z - \beta\delta|e|F_y\tau_y\otimes\sigma_x + \beta_{12}\tau_x\otimes\left(\sigma_x\hat{k}_y - \sigma_y\hat{k}_x\right). \quad (7)$$

When deriving Hamiltonian (7) we have made the four assumptions, which require a more detailed justification.

(i) We have neglected the electric field of the QPC in Hamiltonian H_{SO}. In order to justify this assumption, we have performed additional calculations taking into account the electric field of the QPC i.e., $\partial U_{QPC}/\partial x, \partial U_{QPC}/\partial y$ and we have not observed any influence of this field on the results. This can be understood if we realize that the electric field of the QPC is a few orders of magnitude smaller than the lateral electric field F_y.

(ii) We have assumed the presence of the lateral electric field F_y. This assumption is based on the recent paper, in which Gvozdic and Ekenberg[1] pointed out that in the modulation-doped wide quantum wells, used for the bilayer nanowires fabrication, the large intrinsic F_y exists.

(iii) We have assumed that the intra-subband SO coupling constants $\beta_{11} = \beta_{22} = 0$. This assumption is based on the results of our recent paper[2], in which we have studied the realistic model of the nanowire with the gated $Al_{0.48}In_{0.52}As/Ga_{0.47}In_{0.53}As$ double quantum well. For this nanowire we have determined the SO coupling constants by the self-consistent Schrödinger-Poisson approach. The exemplary results are presented in Fig. 1. Figure 1 shows that the inter-subband SO coupling

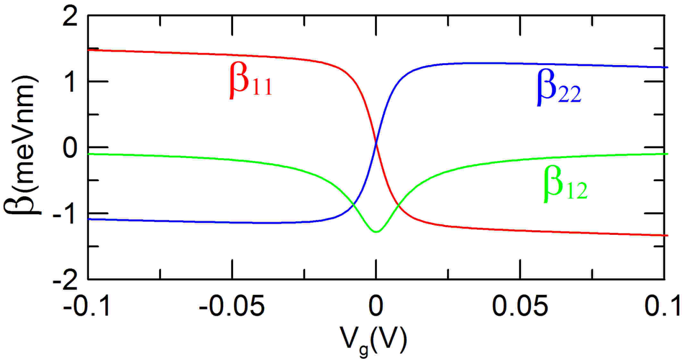

Figure 1. Intra- (β_{11} and β_{22}) and inter-subband (β_{12}) SO coupling constants as a function of gate voltage V_g for the gated $Al_{0.48}In_{0.52}As/Ga_{0.47}In_{0.53}As$ double quantum well structure.

constant β_{12} is an even function of the gate voltage and exhibits the "resonant behavior" at $V_g = 0$ corresponding to the symmetric geometry of the heterostructure. Simultaneously, for $V_g = 0$, the intra-subband SO coupling constants β_{11} and β_{22} change the sign and for $V_g = 0$ are equal to $\beta_{11} = \beta_{22} = 0$. The similar behavior has been recently reported by Calsaverini et. al. for $InSb/Al_{0.12}In_{0.88}Sb$ double quantum well[3]. Therefore, the assumption $\beta_{11} = \beta_{22} = 0$ corresponds to the symmetric geometry of the heterostructure, which is convenient for the studied effect because then the inter-subband SO interaction, which generates the spin filtering effect, is maximal.

(iv) We have neglected the Dresselhaus SO interaction. In our paper, we consider the bilayer nanowire, which can be experimentally realized in the system built from double quantum wells with the central barrier or a wide quantum well, in which the Coulomb repulsion gives raise to the soft barrier in the middle of the quantum well. For such a wide quantum wells needed to fabricate the bilayer nanowires, the strength of the Dresselhaus SO interaction is a few order of magnitudes weaker than the Rashba SO coupling. This results from the fact that the Dresselhaus coupling parameter $\gamma_D \sim \langle k_z^2 \rangle$, and for the infinite quantum well takes the form $\gamma_D \sim 1/d^2$ (d-is the quantum well width in the z direction). To give some concrete example, based of the formula[4]

$$\gamma_{D,v} = \gamma_D^{3D} \langle \hat{k}_z \rangle_v, \tag{8}$$

we have calculated the linear Dresselhaus SO coupling parameters for the realistic model of the bilayer nanowire with the gated $Al_{0.48}In_{0.52}As/Ga_{0.47}In_{0.53}As$ double quantum well[2] (the same structure has been considered for the calculations of the Rashba parameters presented in Fig. 1). Results for $\gamma_D^{3D} = 0.0237$ meVnm[35] are presented in Fig. 2. If we

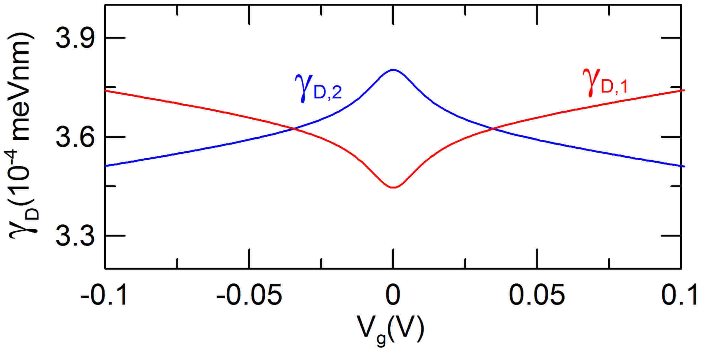

Figure 2. Intra-subband ($\gamma_{D,1}$ and $\gamma_{D,2}$) Dresselhaus SO coupling constants as a function of gate voltage V_g for the gated $Al_{0.48}In_{0.52}As/Ga_{0.47}In_{0.53}As$ double quantum well structure.

compare these results with the Rashba SO parameters presented in Fig. 1 we see that for the considered double quantum well, the Dresselhaus SO coupling constants are four orders of magnitudes smaller than the Rashba coupling constants. This means that the Dresselhaus SO interaction does not affect the spin filtering presented in the paper. In order to support this argument in more quantitative manner we performed the additional calculations with the inclusion of the Dresselhaus term and obtained that $P(E_F)$ presented in Fig. 2(b) in the main paper did not change.

References

1. Gvozdic, D. M. & Ekenberg, U. *Appl. Phys. Lett.* **90**, 053105 (2007).
2. Wójcik, P. & Adamowski, J. *Semicond. Sci. Technol.* **31**, 115012 (2016).
3. Calsaverini, R. S., Bernardes, E., Egues, J. C. & Loss, D. *Phys. Rev. B* **78**, 155313 (2008).
4. Fabian, J., Matos-Abiague, A., Ertler, C., Stano, P. & Zutic, I. *Acta Physica Slovaca* **57**, 565 (2007).
5. Jancu, J. M., Scholz, R., de Andrada e Silva, E. A. & La Rocca, G. C. *Phys. Rev. B* **72**, 193201 (2005).
